# Immediate fall prevention: the missing key to a comprehensive solution for falling hazard in older adults

**DOI:** 10.3389/fnagi.2024.1348712

**Published:** 2024-04-04

**Authors:** Khashayar Misaghian, Jesus Eduardo Lugo, Jocelyn Faubert

**Affiliations:** ^1^Sage-Sentinel Smart Solutions, Kunigami-gun, Okinawa, Japan; ^2^OIST Innovation, Okinawa Institute of Science and Technology Graduate University, Onna, Okinawa, Japan; ^3^Faubert Lab, School of Optometry, Université de Montréal, Montreal, QC, Canada; ^4^Facultad de Ciencias Físico Matemáticas, Benemérita Universidad Autónoma de Puebla, Puebla, Mexico

**Keywords:** falling, fall prevention, immediate prevention, longevity, aging, aging in place, trauma

## Abstract

The world is witnessing an unprecedented demographic shift due to increased life expectancy and declining birth rates. By 2050, 20% of the global population will be over 60, presenting significant challenges like a shortage of caregivers, maintaining health and independence, and funding extended retirement. The technology that caters to the needs of older adults and their caregivers is the most promising candidate to tackle these issues. Although multiple companies and startups offer various aging solutions, preventive technology, which could prevent trauma, is not a big part of it. Trauma is the leading cause of morbidity, disability, and mortality in older adults, and statistics constitute traumatic fall accidents as its leading cause. Therefore, an immediate preventive technology that anticipates an accident on time and prevents it must be the first response to this hazard category to decrease the gap between life expectancy and the health/wellness expectancy of older adults. The article outlines the challenges of the upcoming aging crisis and introduces falls as one major challenge. After that, falls and their mechanisms are investigated, highlighting the cognitive functions and their relation to falls. Moreover, since understanding predictive cognitive mechanisms is critical to an effective prediction-interception design, they are discussed in more detail, signifying the role of cognitive decline in balance maintenance. Furthermore, the landscape of available solutions for falling and its shortcomings is inspected. Finally, immediate fall prevention, the missing part of a wholesome solution, and its barriers are introduced, and some promising methodologies are proposed.

## 1 Introduction

The aging crisis is one of the biggest, unprecedented challenges that humans are on the verge of encountering. There was no time in the history of civilization as we know that life expectancy reached the current average length. Thanks to medical advancements, humans have found solutions to cure or manage numerous fatal diseases and dangerous conditions. On the other hand, technology and innovations found many ways to address scarcities in vital resources like food and living spaces. Therefore, drastic changes in age demographic numbers are the natural ramifications of these phenomena. As a result, the status quo of geriatric care is bound to be challenged and experience serious redefinitions.

The drastic expansion in health, financial, and care gap is an imminent threat to human society with longer life expectancy. Holding the current record of 1 billion, the over-60 age group is on its way to accost the 2 billion record by the year 2050, constituting 20% of the planet’s population. Surprisingly, the fastest-growing age group in the world is also the over-60 age group. The reason is that the current life expectancy in the world pushes beyond 73.2 years, while the number is more than 80 years old for numerous countries. The mentioned high rate imposes some critical challenges; the increase in the years that a person suffers from compromised health, inflating inadequacy of the current financial and pension provisions to support the extra years, and, most importantly, the caregiving gap crisis will be inevitable. For example, according to Japan’s ministry of internal affairs and communications, population projections in Japan show that by the year 2060, the ratio of the (20–64) age group to the 65+ age group is going to be 1.2; rest assured, it will be impossible to maintain enough human resources to provide care as far as the concept of caregiving is characterized in world’s current situation. Therefore, without question, soon, a direct care worker or an unpaid caregiver must take care of more than only one care-seeking older adult whether they choose the aging in place, the preferable choice of living, or residing in an assisted living facility. Therefore, the only way to make this option feasible is to unburden the caregiver with as many technological solutions as possible to assist the older adults with activities of daily living (ADL) and other challenges (D’Aunno, 2019).

The domain that relates existing and emerging technologies to aging needs and objectives is called “Gerontechnology” (Bronswijk et al., 2008). While on a more resolute scale, digital technology revolving around aging goals is referred to as AgeTech. The objectives of AgeTech and how it may serve its stakeholders (such as older adults, care-providing enterprises, and trusted family members) are delineated by prominent organizations like Aging2.0, AGE-Well, and one U.S. National Science and Technology special task force. The results unanimously outline six domains to be addressed (Etkin, 2022):

• Finance
• Health
• Cognitive health
• Social and connectivity
• Mobility and transportation
• Activities of daily living

An older adult not only struggles with being gradually deprived of their once-taken-for-granted privileges but, more importantly, dreads the moment that a trauma like a fall injury takes a massive part of these already-limited necessities away in just a matter of seconds. Therefore, while it takes a lot of strategizing, innovating, and resources to improve even the most minor aspect of the mentioned objectives, one trauma accident like an injurious fall could incur irreversible, significant setbacks on them. Therefore, the mission must be the innovation of an array of solutions that firstly preserve the already existing aspects of the mentioned six domains, then, after, target to improve the aspects of these objectives (Gawande, 2014; Etkin, 2022).

Also, the outline might imply that the objectives above characterize some distinctively divided fronts, but in reality, things are highly interrelated. It is worth noting that these interconnections make every compromise permeate all aspects of a life of an older adult and also signify traumatic accidents like falls as drastic limitation-imposing factors that could put severe strains on the basic livelihood objectives of an older adult (Etkin, 2022).

Many studies aim to demonstrate these interrelations. For example, one study on 976 subjects over 65 years old revealed that the decline in crystallized cognitive intelligence is significantly detrimental to an older adult’s financial wellbeing (Zhou, 2020). Moreover, another study investigated the role of different social participation in preventing cognitive decline, suggesting that different types of social participation may help maintain cognitive abilities differently in different gender groups (Tomioka et al., 2018). On the other hand, one other group finds the combination of social interactions and physical/cognitive activities very effective in preventing cognitive decline (Hikichi et al., 2017). Finally, while convergent evidence demonstrates a significant correlation between cognitive deficit and higher risk and frequency of falling, cognitive training has shown promise in improving key features of mobility, including balance and walking, for healthy older adults and those inflicted by age-related neurodegenerative conditions (Li et al., 2018).

Also, when it comes to performing advanced ADL, an elderly cohort study in Brazil has demonstrated that the mean number of the advanced activities performed by the subjects with cognitive decline was significantly lower than the mean number of activities that the subjects without cognitive decline were able to do (Dias et al., 2015). Most certainly, some proposed solutions could somewhat alleviate the cognitive decline process, but unfortunately, they are incapable of stopping it in a definite manner. Therefore, a vast part of efforts should be dedicated to finding solutions to help older adults navigate the challenges of life despite the deficits in their cognitive abilities.

All the above being said, given how trauma and especially traumatic fall injury, could devastate the state of being of life of an older adult in the most abrupt manner, we find it necessary to take a close look into how the fall problem in the elderly is being tackled and treated. What are the angles to address the issue, and what is missing from the equation. Below we start by introducing falls as the leading cause of traumatic injury then we go over the causes of disruption of balance in older adults: intrinsic and extrinsic. In that vein, we specifically aim to draw more attention to the mostly overlooked cognitive balance restoration mechanisms in the human brain, which affects older adults with different levels of cognitive impairments to a higher degree. So that, later in the paper, we have grounds to suggest how simple cognitive design aids could help with fall prevention. Thereafter, we explore the categories of solutions available to address the issue of falling. However, we particularly underscore the solutions that aim to prevent falls and how the landscape of fall prevention technology is missing a certain class of solutions: a type of preventive solution that could serve as the last line of defense; in a way that predicts the fall in a matter of seconds and launch a timely and effective intervention mechanism. We will refer to this type of fall prevention solution as immediate prevention throughout the present text. Thereafter, we investigate the obstacles to integrating the mentioned class, and finally, we move on with some suggestions as tenable methods for immediate preventive solutions.

## 2 Geriatric trauma vs. geriatric goals

Twenty-five percent of the total trauma admissions in the U.S. are geriatric trauma. Unfortunately, the phenomenon constitutes the fifth leading cause of mortality in the U.S. Fall injuries, followed by motor vehicle accidents and burns, are the United States’ highest-ranked causes of geriatric trauma (Southern et al., 2022). Moreover, data from 2004 to 2017 from Japan Trauma Data Bank (JTDB) also ranks fall accidents as the number one cause of geriatric trauma leading to 7.7% in-hospital mortality in the 65–79 age group and 6.6% in the ≥80 age group (Miyoshi et al., 2020). Additional conditions like compromised functional reserve and polypharmacy increase the mortality rate in geriatric trauma compared to the trauma mortality rate in younger adults (Dreinhöfer et al., 2018; Gerrish et al., 2018). The numbers from geriatric trauma statistics accentuate the exigency of the demand for innovative solutions accordingly.

Geriatric trauma, with falls as its number one cause, threatens all aspects of main geriatric goals. In case of injury, health, wellbeing, capacity to perform ADLs, and mobility would be compromised. On the other hand, if they elude physical injury, fear of falling will follow in approximately one-third of the older adults who experienced falling. As a result, they would somewhat limit their activities and mobility, which could lead to depression and fewer social interactions (Friedman et al., 2002; Mackenzie et al., 2002; Chamberlin et al., 2005; Segev-Jacubovski et al., 2011). Therefore, addressing the threat that falling holds to the aging society is of the highest significance. Undoubtedly, a comprehensive solution to falling and its consecutive problems could extend beyond fall detection and calling for help. Essentially, it must include programs that reduce the risk of falls and solutions that could prevent and intercept them in the event of incidence.

## 3 Basis of falls

**FIGURE 1:**
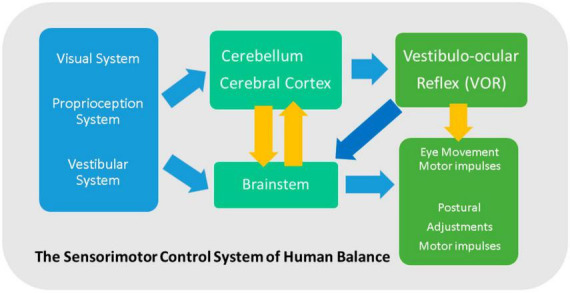
Maintaining balance: integrated sensory input from the visual, proprioception, and vestibular system transmits to the cerebellum, cerebellar cortex, and brain stem. Thereafter, the motor impulses for balance adjustment are sent to the body and eye muscles.

Maintaining balance means keeping the center of mass over the body’s support base. Finding one’s orientation with respect to gravity, having the capacity to determine direction and speed, having a clear vision while moving, and maintaining posture during various actions under different conditions are the features of a well-functioning balance system in humans. It relies on integrated sensory input from the visual, proprioception, and vestibular systems. The visual system provides feedback regarding the uprightness and location of the body in space with respect to objects; the proprioception system communicates pressure and vibratory information from the skin, muscles, and joints; finally, the vestibular system comprises sensing organs that transmit spatial orientation, motion direction, and equilibrium data to the cerebellum, cerebellar cortex, and brain stem. Accordingly, the motor impulses for balance adjustment are transmitted to the body and eye muscles. In other words, maintaining balance is the process of receiving and integrating sensory information from peripheral sources and forming motor output for the eyes and body muscles. Therefore, any originator of neurophysiological changes to the sensory and motor systems, like aging, prompts a greater risk of falling (Figure 1; Vestibular Disorders Association et al., 2016).

Together with the mentioned intrinsic causes of falls, external factors such as environmental and behavioral hazards (i.e., slippery floor, poor lighting, rushing, or wearing improper footwear) compound and elevate the risk of elderly falls (Butler et al., 2014; Appeadu and Bordoni, 2022).

Also, gender is a crucial factor when it comes to the risk of falls and fall-related injuries. Women are at 50% greater risk of falls and non-fatal injuries due to falls. Reduced bone mass density and early osteoporosis due to menopause are believed to be the culprits of more serious injuries after fall incidents (Moreland et al., 2020).

While it is true in general that older women fall more than older men. More detailed research provides a better insight into how falling risk factors pose different threats to different genders. A prominent long-term study of 9 years that captured 1,738 falls from senior living facilities ranked the causes of falls based on the mentioned data acquired from 298 female and 231 male residents (average age of 83), characterizing the most common cause (50%) as incorrect shifts in body weight, 20% loss of support from walkers or similar objects. After that, tripping, bumps, loss of consciousness, and finally, slips were the rest of the causes accordingly. Furthermore, most of the falls happened during walking. Also, while women were more prone to tripping hazards, men were the ones who fell more due to loss of support. Moreover, despite women holding a higher record of fall incidents, men were likelier to fall during sitting-down and standing-up transitions (Yang et al., 2018).

Interestingly environmental hazards are not threatening older men and women the same indoors and outdoors. For that, one study showed that indoor environmental hazards heighten the odds of falling in women compared to older men (Lee, 2021).

Characterizing variation in gait patterns as variations in step width, step length, stride length, step time, stance time, stride velocity, and single support time, one study demonstrated that women have a higher risk of falling due to the 15%–35% higher variations in their gait patterns while they engaged in a simultaneous task during gait (Johansson et al., 2016).

Back to the intrinsic causes, it has been shown that the declines in motor systems manifested in lower and also upper-extremity muscle strength, aerobic endurance, agility, and dynamic balance performance significantly contribute to falling in the aging population (Toraman and Yıldırım, 2010). Whereas these performative changes majorly stem from conditions like Sarcopenia (refers to muscle-mass loss due to aging), Dynapenia (denotes the loss of muscle functionality and strength due to aging), and neuromuscular disorders due to aging. Consequently multiple studies investigated the associations of these factors with falls in older adults (Scott et al., 2015; Yeung et al., 2019; Lim et al., 2020). In that light, a meta-analysis study on databases like MEDLINE, EMBASE, Cochrane, and CINAHL up until May 2018, independent from sex population, continent, and study design, showed that Sarcopenic patients are significantly at a higher risk of falling compared to non-sarcopenic patients (Yeung et al., 2019). Moreover, in a study that categorized elderly falls into two fragile falls (for example, due to leg weakness during changing position) and non-fragile falls (slipping or tripping), Sarcopenia showed a significant correlation with fragile falls. Also, it was identified as a fall risk factor for patients with fragility hip fractures (Lim et al., 2020). Interestingly, in another study, while sarcopenic male patients showed a 2.5 times greater likelihood of falling compared to non-sarcopenic male patients, such association was non-existent in female counterparts (Scott et al., 2015). However, other studies introduced declined hand strength (Sayer et al., 2006; Furrer et al., 2014) and lower extremity function (Cummings et al., 1994; Sayer et al., 2006; Furrer et al., 2014; Scott et al., 2015) as reliable predictors of falls in both sexes, making Dynapenia a preferred predictor of falls and fracture risk in older adults (Scott et al., 2015).

Moving on to intrinsic neurological causes, a multitude of factors could contribute to age-related neurophysiological attenuation. For example, sensitivity loss to visual contrast has been shown to be attributed to higher internal noise due to aging (Allard et al., 2013). Also, the weakened neurohumoral responses due to aging beget delayed and less efficient responses to stimuli (latency). Moreover, co-morbidities like diabetes complications leading to peripheral neuropathy could cause the loss of proprioception, which only means the loss of a whole array of external sensory cues on top of the aforementioned delays and weaker signals (loss of sensory bandwidth) (Southern et al., 2022). Therefore, from a systemic point of view (sensorimotor control system of human balance), the sensory input becomes noisier, its bandwidth decreases, and the latency affects all sensory and motor signal transitioning.

Contrary to what was once presumed, the balance is more than an automatic and low-level task. For years, the consensus was that balance and gait are exclusively automatic, bottom-up, biomechanical motor processes (Segev-Jacubovski et al., 2011). In that light, since most falls occur during walking (Sartini et al., 2010), disturbances in gait were regarded as the leading cause of falls. Therefore, musculoskeletal, cardiovascular, and neurophysiological alterations like visual, vestibular, proprioception impairment, and blunted postural responses due to aging were viewed as gateways to gait and balance alterations that eventually result in falls. All in all, when it came to falling, declines associated with aging were solely associated with disrupting the low-level automatic processes of human gait that lead to falls (Sudarsky, 1990, 2001; Alexander, 1996; Horak, 2006; Segev-Jacubovski et al., 2011). However, later investigations and accumulation of evidence pointed toward a strong association of higher-level, top-down cognitive functions with gait and balance, Including some studies suggesting a possible causal relationship between cognitive function and falls (Segev-Jacubovski et al., 2011). Furthermore, it has been shown that the link between cognitive decline and gait impairment becomes more conspicuous when stabilizing balance in the face of more demanding and challenging situations (Segev-Jacubovski et al., 2011; Allard et al., 2013). That means a thorough analysis of the causes of falls implicates investigating both automatic and cognitive factors.

The annals of fall incidents among older adults with different forms of cognitive decline also highlight the findings above. The probability of falls among older adults with cognitive impairment is twice that among older people with intact cognitive capacities (Eriksson et al., 2008). Additionally, the significantly higher frequency of falls in patients with dementia (80%) who almost have no motor function problems underscores the role of higher-order processes such as cognition and decision-making in maintaining balance (Shaw, 2003; Van Iersel et al., 2006). The connection between gait and cognitive function also gained more traction when some studies suggested that gait utilizes specific cognitive capacities or, on another note, changes in gait could be regarded as indicators of future full-blown cognitive decline (Alexander and Hausdorff, 2008). Therefore, it is only practical to classify the roots of fall into two low-level automatic and high-level cognitive categories.

While the low-level automatic causes of falls are well characterized, interestingly, not all cognitive functions are related to falls. As a matter of fact, studies show no significant difference between general measures of cognitive function (i.e., the Mini Mental-State Examination) and the long-term memory capacity of fallers and non-fallers (Hausdorff et al., 2006; Van Iersel et al., 2006; Herman et al., 2010). However, the performance of fallers in executive function (EF) and attention tasks demonstrate significant inferiority (Hausdorff and Yogev, 2006; Hausdorff et al., 2006; Springer et al., 2006; Holtzer et al., 2007; Liu-Ambrose et al., 2007; Allcock et al., 2009; Herman et al., 2010; Lord et al., 2010). EF entails three cognitive function measures: working memory, cognitive flexibility, and inhibitory control. Additionally, the EF’s cognitive measures have proven reliable predictors to identify prospective fallers, contrary to the other cognitive factors. More distinctively, inhibitory control and decision-making processes are underlined as critical components linking EF to falls (Liu et al., 2014; Bolton and Richardson, 2022). Additionally, it is suggested that monitoring and updating information as special subdomains of EF, are associated with stride time variability in healthy older adults, attesting to the relationship between cognitive functions and gait control (Persad et al., 2008). It is, therefore, more practical to regard the high-level cognitive capacities related to falls in a more specified and narrowed-down manner.

Studying the underlying neuro-cognitive mechanisms, besides understanding which cognitive capacities are critical to balance maintenance, is as essential. When low-level automatic balance-maintaining processes come short of justifying how the human body could recover its stability in the face of high-intensity perturbations, high-level cognitive functions could provide explanations (Dakin and Bolton, 2018). Unsurprisingly, these cognitive functions are mostly to be of predictive nature. A growing body of literature has been looking into the association of the predictive mechanisms that expedite balance recovery with cortical, basal ganglia, and cerebellar networks (D’esposito et al., 1995; Braitenberg et al., 1997; Morasso et al., 1999; Doya, 2000; Müller and Knight, 2006; Rogers and Mille, 2016). Briefly put, cognitive balance restoration involves past-learned experiences and updating postural adjustments according to one’s internal model of the forthcoming disruption (Dakin and Bolton, 2018).

## 4 High-level balance maintenance

As mentioned above, top-down cognitive, balance maintenance and recovery mainly include predictive mechanisms. Some of these processes are known (Dakin and Bolton, 2018):

• Central set
• Dynamic prediction of instability and sway
• Prediction using an internal model
• Affordances for action in balance recovery

A better understanding of these high-level processes along with the automatic low-level balance mechanisms, provides key insights for devising technologies and interventions necessary to protect older adults from falling threats despite the inevitable neurophysiological changes resulting from aging.

### 4.1 Central set

Central set refers to updating (setting) the state of the central nervous system (CNS) using descending commands (neuronal signals from the brain to the neurons in the spinal cord) following the dynamic prediction of instability and sway. The descending commands are expressed dynamically by predicting the aspects of the perturbation. Therefore, shifting the CNS to a new state would prepare many features of a proper response without visible manifestation. Consequently, one prominent effect is the acceleration in generating proper counter-reaction and postural adjustment (Horak et al., 1989). Therefore, any cognitive impairment that disrupts the central set would lead to a smaller window of opportunity in addition to a more extensive effort, given the unchanged state of the CNS.

The influential work of Horak et al. (1989) on the central set has examined and compared the relative influence of the central set and peripheral drive (automatic motor response). In the experiment, a fixed-support platform translation paradigm has been adopted. The objective of the study was to investigate how expectations impact postural responses in humans facing forward sway perturbations to the platform. The extent of in-advance information about the speed and the size of the forthcoming perturbation was controlled so subjects could experience different central set conditions. More precisely, the tests were conducted with varying familiarity with the stimulus velocities or amplitudes under expected and unexpected scenarios.

Interestingly, findings revealed that the anticipation of postural stimulus amplitude significantly influenced initial torque responses, leading to subjects over-responding to larger perturbations and under-responding to smaller ones. Contrarily, the expectation of postural stimulus velocity had a lesser effect on initial torque responses, leading subjects to consistently over-respond when the perturbation velocity was unexpected. Central set conditions did not affect initial response latencies or the relative onset of activations of corresponding muscles. Additionally, errors in initial response magnitude due to central set effects were partially corrected by reciprocal activation. Highlighting how the central set, involving expectations of stimulus amplitude and velocity, influences postural responses, Horak et al.’s (1989) study underscores the interplay between cognitive processes related to the central set and automatic postural responses in maintaining balance and stability.

Several EEG studies have demonstrated that on the central region of the scalp, a slow wave potential emerges and starts and continues to build up right until the perturbation strikes. At this point, a different cortical potential (N1 response) conjoins the activities as a post-perturbation potential (Adkin et al., 2008; Jacobs et al., 2008; Mochizuki et al., 2008).

Later it was shown that the anticipatory cortical activity has similar qualities whether the disturbance is internal or external and scales with the amplitude of the forthcoming perturbation (Mochizuki et al., 2009, 2010). More importantly, activity changes were observed and recorded simultaneously with these anticipatory cortical activities in the spinal cord circuitry (Wälchli et al., 2017). The above could imply that these cortical potentials are manifestations of cortical processes that adjust the central set (Jacobs et al., 2008). However, the causality of this relationship is yet to be investigated (Dakin and Bolton, 2018).

### 4.2 Dynamic prediction of instability and sway

In a scenario where an external perturbation induces a stepping action, once older individuals with a higher risk of falling detect a postural threat, they tend to step earlier than young low-risk adults, even though the perturbation could have been managed by using a fixed-support reaction. The early stepping could signify disruptive sensory signaling that leads the high-risk individual to resort to a default strategy without scaling, incoherent to the perturbation, and might involve extra follow-up compensations (Rogers and Mille, 2016). As mentioned before, somatosensory/proprioceptive, visual, and vestibular systems play the primary role in the sensory signaling of balance (Peterka, 2018). In addition to their role in automatic balance recovery, sensory signals provide real-time predictive cues for the CNS to decide whether to intervene. Therefore, inconsistent sensory signaling with latency results in an obsolete real-time estimation of body position, causing the mentioned scenario above (Dakin and Bolton, 2018).

### 4.3 Prediction using internal model

Forward models are a type of internal model in the brain that maps a movement to an expected sensory consequence. These neural substrates are deemed to be the basis of supervised learning mechanisms in the cerebellum (Flanagan and Wing, 1997; Pasalar et al., 2006; Popa et al., 2012; Brooks et al., 2015). The difference between the expected sensory consequence signal and the actual sensory feedback could characterize the compensation to a perturbation or any other stimulus. The error signal could serve to calculate necessary postural compensation or update the corresponding forward model (Flanagan and Wing, 1997). On the other hand, one other type of internal model called the inverse model receives sensory predictions and generates motor commands. When coupled with a forward model, the combination could provide motor compensation for predicted states of the body. In fact, as a general model for sensorimotor learning and control, it could simply explain the mechanics of predicting postural disturbance and the generation of a suitable compensatory response (Wolpert and Kawato, 1998; Haruno et al., 2001, 2003). Interestingly, it is the consensus that the basis of the evolution of internal models was to counteract the neural conduction latency in the larger bodies, which signifies the threat that latency could pose in balance recovery scenarios (More et al., 2010).

### 4.4 Visual priming based on affordances for balance recovery

The concept of “affordances” states that our brain constantly evaluates our environment and encodes this information to the set of movements that could be performed in that environment. In other words, it assesses if the current environment at any given time could afford the type of movements we might want to perform (Buccino et al., 2009; Cardellicchio et al., 2011; Makris et al., 2011; Franca et al., 2012). Canonical cortical neurons, a class of neurons found in the brain’s cerebral cortex, are characterized by their ability to respond to multiple sensory modalities. They possess the unique property of responding preferentially to different modalities of multisensory sensory inputs and are identified as the neurophysiological substrate of the affordance effect in monkeys. However, while behavioral studies supported the existence of the affordance effect in humans, neurophysiological investigations demonstrated contradictory results regarding neural substrate identification (Franca et al., 2012). Moreover, mentioned human studies show that in the absence of movement, a measurable link exists between the observation of a simple object and cortical motor activation (Franca et al., 2012). Therefore, visual priming based on affordances is a predictive mechanism that most certainly plays a significant role in postural balance control (Dakin and Bolton, 2018).

All the cognitive functions above heavily rely on consistent and undelayed sensory input. The central set hinges on a persistent and redundant flow of sensory information to make stabilization more efficient with less exertion. Disruptive and delayed sensory input sends faulty cues to CNS, which results in incorrect estimation of body position and inconsistent motor response. Furthermore, the error signal critical for estimating motor compensation and tuning the forward models will be drastically compromised due to the asynchronicity between the expected sensory signal and the actual noisier and delayed sensory feedback. Finally, the estimation of affordances would be outdated and less accurate. Let us not forget that the problems above do not factor in other defects in cognitive functions due to aging or cognitive decline. Therefore, finding a comprehensive solution for falling prevention is not only very complicated but also very subject-dependent. However, on a more fundamental level, interventions that alleviate common risk factors could have a significant impact. For example, given the significance of sensory input, any solution for a stronger sensory signal to noise, overcoming latency, or providing a wider sensory bandwidth to a person with a higher risk of falling could save them from falling and its dire consequences.

## 5 Falling solutions by the present-day technology

Given the scale of the fall threat, the contemporary repertoire is not the perfect responsive bundle. In 2022, more than 200 companies and startups offered services and products addressing the six domains of aging challenges. Given the amplitude of the menace that geriatric trauma (especially falling) poses to all aspects of aging goals, some initiatives aimed to address the falling problem through the following categories:

• Fall detection and calling for help and assistance
• Balance training to reduce the falling risk factors (long-term prevention)
• Functional fitness training to improve balance and strength, and flexibility (long-term prevention)
• Hip protectors, as wearable garments designed to protect the hip using plastic or foam padding materials
• Wearables airbags to reduce the impact of falling

Fall detection and alerting solutions could efficiently reduce the dire consequences of the fall due to speeding the rescue process, and as a result, created one of the most saturated markets in the geriatric technology sector (Etkin, 2022). Unfortunately, they cannot prevent the hip from impact and fracture. Furthermore, balance training and functional exercise solutions drew significant attention due to their adequate capacity to improve motor response for balance recovery and, potentially, cognitive functions (Etkin, 2022). Without a doubt, preventing falls is the most optimal resolution to fall-related fractures and injuries. Accordingly, in a thorough review, researchers demonstrated that physical therapy in the form of a combination of strength and balance training is the most effective way to prevent falls in long-term care facilities for older adults. Also, in the case of frail older adults with a higher risk of falling, environmental hazard assessment and modification combined with the utilization of hip protectors proved to be beneficial. However, the authors concluded that the Ideal approach is to tailor a multifaceted intervention program specific to every individual (Karinkanta et al., 2010). Evidently, individually tailored solutions usually are not very cost-effective and maintaining their consistency could be challenging. Not to mention that the change of habits and lifestyle for older adults is one farfetched objective.

Moreover, classic hip protectors and later airbag solutions gained some popularity due to their simplicity and reliability. However, wearing these apparels proved to be cumbersome. Also, in the case of airbag solutions, there is a chance that the technology could backfire and only change the form of injury.

In addition to available solutions on the market, one thorough review on the effect of cognitive therapy on falling risks highlights the up-and-coming applications of using multimodal cognitive training and its incorporation in clinical practice for fall risk reduction in patients with cognitive impairment or age-related frailty (Segev-Jacubovski et al., 2011).

Regardless, despite the effectiveness of all the solutions mentioned above, appending a less complex and low-maintenance solution as another layer of safety that could intercept the falls seems extremely essential. While this last layer of safety could be devised using modern-day technology, it is crucial that its functionality would be simple, robust, and seamless and does not impose extra levels of complexity on the stakeholders.

Again, given the inevitable nature of neurophysiological changes or, in many cases, cognitive decline, solutions that could immediately intercept and prevent falls are missing from a comprehensive fall solution collection (Table 1).

**TABLE 1:** While categories like fall detection and calling for assistance, long-term fall prevention, airbag wearables, and shock-absorbing flooring constitute a highly saturated market, solutions to address immediate fall prevention are notably missing from the equation.

Fall detection/call for help (post-accident)	Long-term Fall prevention: balance training (BT) Functional fitness training (FFT)	Hip protectors/wearable airbags	Immediate fall prevention
>6 commercialized solutions	>7 commercialized solutions	>8 commercialized solutions	None

## 6 Hip fracture, the most unwelcomed aftermath

Hip fracture is among the most insidious consequences of falling in older adults. In fact, 95% of hip fractures are due to elderly fallings. Unfortunately, full recovery odds are not very high, and the aftermath comprises pain, immobilization, and loss of many aspects of the patient’s independence or death (McMillan et al., 2012). Therefore, studying the biomechanics of hip fractures due to falling could provide a great deal of insight for developing prospective solutions to prevent hip fractures. In that light, one study aimed to determine the factors that differentiate the falls that lead to hip fractures from other falls by investigating the largest existing dataset of real-life fall videos (2,377 falling incident videos) (Yang et al., 2020). The study demonstrated that the biomechanics of the fall could play as important of a role as bone density. Notably, while a bone density decline of one standard deviation doubled or tripled the odds of fracture risk, sideways falling and landing on the hip multiplied the fracture risk by 6 and 30 times, respectively.

On the other hand, falls that involved landing on hands, grabbing, or hitting some object that lessened the impact rarely resulted in hip fractures. However, in the study, the categories of fall characteristics were limited to the direction and height of the falls and their point of impact, which was recorded through questionnaires answered by fallers themselves or witnesses. Furthermore, the effect of wearing hip protectors (a garment designed to protect the wearer from hip fracture using padding materials) was investigated. Seventy-three percent of the fallers were equipped with hip protectors, and it was found that the risk of fracture was significantly lower if the hip protector was worn (Yang et al., 2020). Beyond any doubt, the mentioned results could play a prominent role in designing any fall prevention solutions ranging from balance and functional training for fall prevention to hip protectors or inflators.

## 7 Prospective immediate fall prevention solutions

Above, we have explored how a healthy brain uses automatic peripheral balance adjustments and high-level predictive mechanisms to maintain balance. Later, we underscored the effects of neurophysiological alterations due to aging (noisy and latent visual, proprioception, and vestibular systems, along with slower neurohumoral activation and loss of sensory bandwidth due to co-morbidities) that directly disrupt both automatic and cognitive mechanisms of balance restoration (Southern et al., 2022). Furthermore, we elaborated on how any level of cognitive attenuation in certain aspects of cognitive function could compound fall risks. For example, impairment in EF would directly disturb cognitive processes, such as central set, dynamic prediction of sway, internal prediction model, and affordances for action, which are believed to be the optimal mechanisms for balance recoveries against high amplitude balance perturbations (Dakin and Bolton, 2018). Therefore, the prospective solutions aiming to intercept and prevent falling immediately must, at least partially, if not completely, compensate for the drawbacks of the mentioned deficiencies.

**FIGURE 2:**
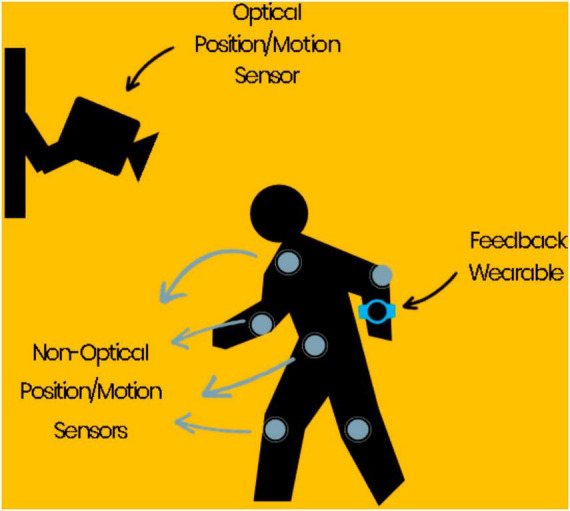
An example of an immediate fall prevention solution: an array of optical and non-optical position and motion sensors along with algorithms that could predict a fall a few seconds before the onset, followed by timely feedback to the wearables.

In that light, one plausible solution is to combine optical and non-optical real-time position and motion sensing with algorithms that could predict a fall before the onset, followed by timely biofeedback. Such a system could function as an auxiliary to the compromised automatic and cognitive mechanisms as follow: circumventing the consequent delays in the visual, vestibular, and proprioceptive sensory input, expanding the sensory bandwidth by sensing independently in parallel and also, providing more time for the now-slower neurohumoral activations through early alarming (Figure 2).

All in all, an effective immediate prevention solution entails two critical parts: firstly, prediction of the accident in a matter of seconds; secondly, an efficient intervention mechanism to intercept the accident within a few seconds window of opportunity. Undoubtedly, solutions of this nature should address privacy concerns, the loss of sense of independence, and adoption dispositions as the biggest challenges of these kind of technologies in order to become practical. Nevertheless, prospective algorithms for predicting the onset of falls are an imperative part of a pragmatic, immediate fall prevention solution, and evaluating them is merely necessary.

### 7.1 Barriers, contingencies, and proposed methodologies

As mentioned above, an immediate preventive solution has two essential parts: prediction and intervention. As for the prediction, the latest machine learning methodologies indeed harbor much promise. However, regarding the prediction of falling, some problems exist that hinder us from profiting fully from some of the contemporary machine-learning approaches. As a consequence, while almost all proposed machine learning methods demonstrate decent performance for only detecting falls, performances by the mentioned algorithms for predicting the falls before occurrence offer diminished promise. Fast-forward to modern machine learning techniques, a convolutional neural network (CNN) is a class of artificial neural networks (ANN) comprised of many layers (deep neural network) best known for but not limited to, its application in image and video processing. The main demarcating feature of CNN is its ability to encode feature maps by sliding convolution kernels along the input features (from the input image or previous layers) and applying nonlinear operations to them (Mouton et al., 2021). Regarding falling, all proposed CNN-based approaches revolve around learning patterns from image datasets of falling images (Li et al., 2017; Tsai and Hsu, 2019; Yhdego et al., 2019; Casilari et al., 2020).

In the meantime, recurrent neural networks (RNN), famous for sequential applications like speech recognition, is a class of ANN that allows for connections between output and input nodes so that the output could affect the input of the related node in the following sequence. In other words, the network possesses a temporal dynamic. Furthermore, incorporating memory (internal states) allows the RNN to process variable-length sequences (Dupond, 2019). The long short-term memory (LSTM) network is a well-known RNN network with the mentioned internal states (Hochreiter and Schmidhuber, 1997). Regarding falling, some studies resorted to combining the sequential learning power of LSTMs with the CNNs’ high capacity for pattern learning to include the spatiotemporal information that lies in the falling sequences (Zhang J. et al., 2020). However, given that CNN and RNN approaches are both supervised methods, good results are highly dependent on having access to rich datasets that contain thousands of videos of natural falling, preferably in older adults. On the other hand, gathering many videos of older adults falling is a challenging and arduous objective. For example, it took 10 years to capture 2,377 falls from 646 residents, which constitutes the largest database for falls among the elderly so far (Yang et al., 2020). Moreover, although unsupervised methods like autoencoders could identify anomalies in the incoming input, their best performance is limited to detection.

Nevertheless, while almost all the disposition to solve the fall problem is toward deep learning methods, it may be time to look into other methodologies.

### 7.2 Prospective methodologies

Regarding the prediction part of an immediate prevention solution, below, we propose two approaches that bear a great deal of promise: the Swiss cheese model and biological motion detection.

#### 7.2.1 The Swiss cheese model

The Swiss cheese model is helpful in risk management and analysis as a model of accident causation. It is used in risk analysis and management, including aviation safety, engineering, and healthcare. In the Swiss cheese model, an organization defends itself against failure by barricading itself with imperfect barriers. A barrier and its flaws are characterized as a slice of Swiss cheese with holes representing the weak points in that barrier. The holes are assumed to continuously vary spatiotemporally in the respective barrier (Swiss cheese slice). The failure occurs when a hole from all slices aligns at any given moment allowing a tentative hazard to pose a threat to the system via its so-called trajectory of accident opportunity (Symon and Wilson, 2002; James et al., 2005; Stranks, 2007).

The Swiss cheese model further explains that while anticipating all possible scenarios of accidents is not plausible, knowing the dynamics, interrelationship, and latent conditions of the purported holes can be used to avert their alignment and the consequential failures of the system. In their study of 84 severe maritime accidents, after classifying the accidents into six categories, Fukuoka and Furusho (2016) determined the relationship between latent conditions and hole characteristics. In the model, the safety management system (SMS) in organizations and risk management at local workspaces were characterized as defensive layers. Moreover, 10 latent conditions were defined by modifying the software-hardware-environment-liveware (SHEL) model (Fukuoka and Furusho, 2016, 2022). Results demonstrated that in all categories, except in the sinking cases, the holes mainly exist in the SMS defensive layer.

Moreover, in cases involving collisions, occupational casualties, fire, or explosion, holes in the defensive layer of risk management are common in the early stages of the risk management process. The most common latent condition was an inadequate operator condition. So, these findings suggest that the locations and causes of hole openings can be determined. Consequently, accidents can be systematically avoided by applying methods for closing the holes using the results of this study.

The SHEL model has applications in plane crash prevention and can be adapted for other forms of accidents, like university laboratory accidents (Fukuoka and Furusho, 2022) or human falls. Clearly, since the operating environment of each accident may differ, details for a different type of environment must be modified and adjusted accordingly (Hawkins and Orlady, 2017). For example, in the case of university lab accidents, the SHEL model approach for accident prevention includes the following:

1. **S** stands for software and refers to the procedure and rules in place during the accident.
2. **H** stands for hardware, which includes the machines/equipment/facility state and the human-machine interface.
3. The environment is represented by **E**, including workplace environmental conditions, site traffic dynamics, and geographical features.
4. **Lc** is the Central Liveware and refers to the cause of the operator’s accident. Physical and sensory limits, physiological and psychological conditions, workload management, knowledge, skills, experience, and training are all subsets of **Lc**.
5. **Lp** is the Peripheral Liveware, which is positioned around **Lc**, including the following aspects: communication among participants in experiments, teamwork, near-miss response, experiment discontinuation criteria, the safety management system status, the environment itself, and external organizations that affect university safety.

#### 7.2.2 Prediction using biological motion detection

Biological motion perception is essential not only for survival but also for human social life. Identifying the neurobiological substrates has long been a topic of inspection. While it is widely presumed that the integration of dynamic form cues and local motion in the brain gives means to the visual system to perceive biological motion stimuli, recent research has highlighted the importance of dynamic form cues in such a process. Furthermore, inspired by previous neurophysiologically plausible biological motion perception models, a descriptive risk-averse Bayesian model capable of discerning a ball’s direction from a set of complex biological motion soccer kick stimuli is proposed (Misaghian et al., 2022a,b,c). Acknowledging the two-stream theory, the model only represents the dorsal pathway as the visual system’s motion information processing section. The human behavior data and stimuli for this model were obtained from a previous psychophysical study on athletes (Romeas and Faubert, 2015). The mentioned model successfully managed to simulate the psychometric function of athlete subjects, and the correlation analysis reveals a significant and robust correlation between the model and human behavior. By relying solely on motion information processing, the results support the theories favoring motion cue importance over dynamic form by testing biological motion perception (Misaghian et al., 2022b).

The model, as mentioned above, could be adapted for predicting human falls. Only this time, instead of learning the subtle movements of a soccer kick, it learns to recognize the subtle movements that lead to the onset of falls. That is, the algorithm should be capable of distinguishing when the walk-fall transition is going to happen just by the way the human moves. Nevertheless, there are a few hurdles to achieving such a goal; one is the prediction time before the fall accident happens. Therefore, the algorithm should predict the onset early enough to create a window of a few seconds that allows immediate intervention.

The second problem is the scarcity and sparsity of available data on gait-fall transitions. For instance, the recorded data containing the footage of falls in older adults are significantly limited. As a consequence, the situation not only calls for the demand for algorithms that could perform successfully with limited data but also an increase of awareness on this issue, close collaboration of researchers and public and private health institutions to collect more and better data to further the performance of algorithms of this kind and deep neural network models.

## 8 Discussion

For the prediction part, the Swiss cheese model and biological motion detection approaches retain qualities that align with the requirements of a comprehensive immediate fall prevention solution. Qualities like the capacity to be uniquely designed for individuals (the Swiss cheese model) or being less data-hungry like supervised deep neural networks (biological motion detection). Regardless, the prospective approaches are not limited to these examples, and searching for such approaches must become a subject of interest in the research communities. The importance of such algorithms becomes more incandescent when we consider these algorithms as means that could bridge the gap between the shortage of data and deep neural network solutions by creating a sustainable infrastructure for gait-fall transition data acquisition. So, the problem could benefit from the power of these modern algorithms.

The design of the intervention part of a comprehensive immediate fall prevention solution could encompass a great range from sensory feedback to verbal instructions and mechanical interceptions by robots or inflatable safety belts. However, the design indeed hinges on the performance and the nature of the output of the prediction part. For example, a wearable that could communicate special sensory feedback that alerts and expedite motor activation sounds plausible for a prediction method that predicts the onset in about 3 s before the onset. In the meantime, an intervention through verbal instructions requires more time and will not be a good fit.

Moreover, the design of the intervention must address various groups of at-risk populations. Clearly, a design to intercept falls immediately in a healthy aging population will probably not be as effective for the aging population with neurodegenerative conditions such as dementia and Parkinson’s disease. For example, in the dementia scenario, one tactile feedback on the wrist will probably fail to alert the person (Zhang Z. et al., 2020), or in Parkinson’s, even if the subject gets alerted, they might not possess enough motor response resolution to react properly (Dubbioso et al., 2019). Furthermore, it is established that the population with intellectual disabilities (ID) suffers from a concerning rate of falls due to the early onset of aging including highly frequent comorbidities such as polypharmacy, epilepsy, urinary incontinence, and lower levels of physical activity (Blomqvist et al., 2013; Pal et al., 2014; Salb et al., 2015; O’Dwyer et al., 2016; Molina-García et al., 2018). Unfortunately, while the ID population sustains a lower balance and self-efficacy than the general population, the research on their fall prevention programs is limited (Choi, 2021). Nevertheless, it is well-known that any feasible intervention addressing the population involves implementing different prospects of the social cognitive theory. The social cognitive theory proposes that the interplay between personal and environmental factors and behavior itself shapes one’s actions and behavior. This theory has been introduced as a useful framework for identifying fall risk factors and developing intervention paradigms for such disabilities (van Schijndel-Speet et al., 2014; Choi et al., 2020; Choi, 2021).

All in all, many uncharted areas could prove to help solve the immediate trauma prevention problem in older adults. Nevertheless, what is crucial is understanding the criticality of the need for immediate prevention solutions that are currently missing from the solution spectrum due to their complicated nature and the mentioned considerable number of barriers. Moreover, prioritizing immediate prevention development is sustainable in numerous ways. Lowering the cost of medical services through prevention instead of treatment, facilitating long-term care for older adults, reducing inequalities for individuals without financial support for medical treatments and hospitalization, and eventually having sustainable cities and communities that foster the abilities of the older population are just a few examples (World Health Organization, 2021; United Nations, 2023). With that in mind, one primary focus must be providing innovations that could counterbalance the evergrowing and inevitable neurophysiological changes and cognitive deficits leading to problems like geriatric trauma, including fall injuries.

## 9 The future: robotics and quantum computing

The radical advancements in the fields of robotics and quantum computing apprise a new wave of technology and toolsets that will affect many aspects of human life and help with the innovations that communities are longing for to solve substantial problems like global warming and aging. Therefore, it is indeed beneficial to investigate how these fronts could contribute to older adults’ lives.

Robotics could be a great help for studying aging (gerontology) and solving the problems and concerns that come with it. Below are listed some examples that target the primary objectives of AgeTech (Pu et al., 2019; Kulpa et al., 2021; Bardaro et al., 2022; Dino et al., 2022):

• Assisting with ADLs: robots may be designed to assist seniors with ADLs such as daily cooking, cleaning, and medicine administration. The type of assistance that can help seniors preserve their independence and actualize aging in place.
• Companionship: robots may be taught to hold dialogues and give social interaction, which can assist older people in overcoming loneliness and social isolation.
• Monitoring health: Some robots have sensors that can monitor the health of older persons, such as monitoring vital signs or detecting falls. Such robots can alert caretakers if there are any difficulties.
• Rehabilitation: robots can aid older adults with physical therapy and rehabilitation by delivering exercises to help them retain strength and mobility.
• Improving cognitive function: robots may give cognitive stimulation, such as games and puzzles, to help keep older adults’ minds active and engaged.

On the other hand, quantum computers are a form of computer that stores and processes data using quantum bits or qubits, dissimilar to classic computers, where bits are used for processing and storage. However, unlike bits, which may only be in one state (either a one or a zero) at a time, qubits can exist in several states simultaneously. Because qubits have this feature, quantum computers can do some computations significantly quicker than regular computers. However, quantum computers are still in their nascent stages and are not yet generally available. They are also far more complicated to design and run than ordinary computers and prone to mistakes due to the sensitivity of quantum states. Despite these obstacles, researchers and companies all over the world are working to develop and improve quantum computers, which have the potential to solve specific problems much faster than traditional computers and could have a wide range of applications, including medicine, finance, materials science, and molecular biology (Outeiral et al., 2021).

Quantum computing could be utilized in computational biology to solve many biological problems, including aging. Furthermore, quantum computing could help computational biology solve generic data modeling and storage problems on a whole new and scaled level. Below are just a few applications of quantum computing that could revolutionize the field of biology and aging (Kassal et al., 2008; Wiseman and Milburn, 2009; Aspuru-Guzik and Walther, 2012; Biamonte et al., 2017; Fedorov and Gelfand, 2021; Veis, 2022):

• Drug discovery: quantum computers may simulate complicated biological systems like proteins, allowing researchers to develop new treatments and understand how they will interact with the body.
• Genetic analysis: quantum computers may be used to analyze vast volumes of genetic data, allowing researchers to understand better the links between genes, aging, and illnesses and uncover possible therapeutic targets.
• Medical imaging: quantum computers may be used to process and interpret medical pictures, such as CT scans and MRI scans, which can aid clinicians in illness diagnosis and therapy planning.
• Quantum computers may be used to do machine learning activities such as analyzing vast volumes of data to detect patterns and trends, which can be beneficial for jobs such as forecasting the chance of contracting a specific disease.

Overall, the impact of quantum computing on biology, including the biology of aging, is anticipated to be enormous, as it has the potential to expedite scientific discovery while improving prediction and diagnosis accuracy on unprecedented levels. Again, despite all the advancements, it takes a clear, thorough, and constantly challenged roadmap to appropriately address all aspects of aging and achieve wellbeing and longevity.

## Data availability statement

The original contributions presented in this study are included in the article/supplementary material, further inquiries can be directed to the corresponding author.

## Author contributions

KM: Conceptualization, Investigation, Writing – original draft. JL: Conceptualization, Writing – review & editing. JF: Writing – review & editing.
